# Persistently high psychological well-being predicts better HDL cholesterol and triglyceride levels: findings from the midlife in the U.S. (MIDUS) longitudinal study

**DOI:** 10.1186/s12944-017-0646-8

**Published:** 2018-01-03

**Authors:** Barry T. Radler, Attilio Rigotti, Carol D. Ryff

**Affiliations:** 1 University of Wisconsin-Madison Institute on Aging, 2245 Medical Science Center, Madison, WI 53703 USA; 2 Departamento de Gastroenterología, Pontificia Universidad Católica, Marcoleta #367, interior, Santiago, Chile

**Keywords:** Psychological well-being, Lipids, Longitudinal study, Cumulative trajectories, Biomarkers, MIDUS, HDL cholesterol, LDL cholesterol, Triglycerides

## Abstract

**Background:**

Psychological correlates of blood lipid levels have been previously evaluated mostly in cross sectional studies. However, prospectively measured psychological factors might also predict favorable blood lipid profiles, thereby indicating a healthy mind/body interplay that is associated with less disease, better health and longer lives.

**Methods:**

This paper examined whether longitudinal profiles of psychological well-being over 9–10 years are predictors of blood lipid profiles. Using the MIDUS (Midlife in the U.S.) biological subsample (n = 1054, aged 34 to 84, 55% female), cross-time trajectories of well-being were linked with three lipid outcomes (i.e., HDL cholesterol, triglycerides, and LDL cholesterol), measured for the first time at the 2nd wave of the study.

**Results:**

Most adults showed largely stable profiles of well-being, albeit at different levels. Some showed persistently high well-being over time, while others revealed persistently low or moderate well-being. After adjusting for the effect of demographics, health behaviors, medications, and insulin resistance, adults with persistently high levels of environmental mastery and self-acceptance—two components of psychological well-being—had significantly higher levels of HDL as well as significantly lower levels of triglycerides compared to adults with persistently low levels of well-being. Converging with prior findings, no association was found between well-being and LDL cholesterol.

**Conclusions:**

Over 9–10 years, persistently high levels of psychological well-being measures predicted high HDL cholesterol and low triglycerides. These findings add longitudinal evidence to the growing body of research showing that positive psychological factors are linked with better lipoprotein profiles. A better blood lipid profile, particularly higher HDL-C, may be key in mediating how psychological well-being positively impacts health and length of life. Additional research is required to further validate this hypothesis as well as to establish potential underlying mechanisms.

## Background

Psychological correlates of lipids have been studied for several decades, beginning with “occasionally significant” negative correlations between serum cholesterol levels and reported moods, motivation, and arousal [1]. Subsequent studies linked cholesterol to mental disorder [2], depression [3], and anxiety disorders [4]. Recent findings from MIDUS study (Midlife in the United States), a national study of adults aged 25 to 74 at baseline, linked higher levels of optimism (after adjusting for numerous covariates) to higher HDL-C (High Density Lipid Cholesterol) and lower triglycerides, but not to LDL-C (Low Density Lipid Cholesterol) [5]. Such research adds to the growing body of literature linking positive psychological functioning to reduced inflammatory markers and better glycemic regulation [6–8] as well as decreased risk of cardiovascular disease [9], lower risk of disability and cognitive impairment [10, 11], greater resistance to infectious illness [12], and better health and longer lives [13–15].

The present investigation used a theory-based formulation of “eudaimonic” well-being [16], derived from the integration of clinical, developmental, existential and humanistic psychology. The model includes multiple aspects of well-being: autonomy, environmental mastery, personal growth, positive relations with others, purpose in life, and self-acceptance. To date, over 500 publications have linked eudaimonic well-being to life course development and aging, work and family life, and health [17]. However, few of these prior investigations have used longitudinal assessments of well-being. Evidence over a 9–10 year period from the MIDUS national sample revealed that most adults did not reliably increase or decrease (using the index of reliable change [18]) on any dimension of well-being over time [19]. Such stability was notably differentiated, however, with regard to level: some individuals showed persistently high levels of well-being across time, while others showed persistently low or moderate levels. Such trajectories underscore distinctions between *cumulative psychological advantage or disadvantage across time.*

In the present inquiry, we tested whether those with advantaged (i.e., persistently high) trajectories of well-being would have better blood lipid profiles compared to individuals with disadvantaged (i.e., persistently low) trajectories of well-being. Why lipids? Broad evidence has demonstrated a key role of high serum LDL-C levels on hard clinical outcomes due to atherosclerotic cardiovascular disease [20], the main cause of morbimortality worldwide. State-of-the-art care, including high-dose statins to reduce LDL-C, can attenuate the risk of long-term clinical events derived from this disease, however patients still exhibit a high rate of cardiovascular events [21, 22]. Atherogenic dyslipidaemia, defined as an imbalance between proatherogenic triglyceride-rich lipoproteins and antiatherogenic high density lipoproteins, constitutes a key contributor to this residual cardiovascular risk [21–24]. Thus, a first key objective was to examine whether those with longitudinally advantaged trajectories of well-being would have better blood lipid profiles, a question with important preventive significance, given the above clinical literature.

Further, prospective biomedical studies have shown that high levels of HDL-C are powerful negative independent predictors of coronary heart disease (CHD) and stroke [25]. Cohort studies have also linked low HDL-C with memory decline [26] and dementia [27], whereas increasing HDL-C levels have been linked to longevity [28]. A relevant question is whether prospectively measured psychological factors might predict favorable blood lipid profiles, particularly higher HDL-C, thereby implicating salubrious mind/body interplay that is associated with less disease, better health and longer lives.

To reiterate, we used data from a national longitudinal study of U.S. adults, which collected biological measures on a subsample of respondents at the 2nd wave. Building on the evidence of long-term stability in eudaimonic well-being over a 9–10 year period, we tested whether those who showed persistently high (cumulatively advantaged) well-being over time would have better lipid profiles (higher levels of HDL-C and lower levels of triglycerides and LDL-C) compared to adults with persistently low (cumulatively disadvantaged) well-being. If HDL is a “recipe for longevity” [25], our question was whether long-term positive psychological factors might, in turn, predict better lipid profiles.

## Methods

### Sample

The Midlife in the United States (MIDUS) study was initiated in 1995 to better understand connections between psychosocial factors and health in adults aged 25 to 74. More than 4000 respondents were recruited by random digit dialing with oversampling in select metropolitan areas. Twin pairs and siblings of some respondents were also recruited for a total baseline sample of 7108 subjects. A longitudinal follow-up was conducted 9–10 years later and obtained a 75% retention rate, adjusted for mortality (see [29] for details on attrition).

The present investigation is based on a subsample of 1054 respondents, including 388 twins, from the longitudinal follow-up who participated in a biomarker project. All of these individuals had completed two prior waves of psychosocial assessments and were healthy enough to travel to a clinic for biomedical data collection. The biomarker subsample was comparable to the larger MIDUS sample on most sociodemographic characteristics (age, gender, marital status, income) although it was somewhat better educated [30]. Nonetheless, there was variability in educational attainment: 24% had a high school education or less, 52% had some college, and 24% has a college degree. Biomarker respondents were also similar to main sample respondents on health characteristics (subjective health, chronic conditions, health symptoms, body mass index (BMI)).

### Demographic, biomarker and health measures

A number of demographic and health variables (from the Time 2 survey) were examined as control measures. Demographic factors included age (continuous), gender (0 = male, 1 = female), and educational level (from 1 = no school to 12 = PhD, MD, or other professional degree). Medication and health status included: percent of cases who reported taking blood pressure, cholesterol, cortisol, or depression medications at the time of the biological assessments, and a log-transformed measure of insulin resistance. Health behaviors included: number of alcoholic drinks consumed in the past month; current smoking status; a measure of regular weekly exercise; an index of healthy eating (average number of weekly consumption of fruits/vegetables and whole grains, and a white-to-red meat index), and BMI.

Psychological well-being (PWB) was measured with six scales [16]: autonomy, environmental mastery, personal growth, positive relations with others, purpose in life, and self- acceptance. Each scale consisted of three items, with a mix of positive and negative items. Respondents indicated the extent from 1 (strongly agree) to 7 (strongly disagree) to which the statements described them. Negative items were reverse coded so that higher scores reflected more positive appraisals. Summed scores were created from all scales.

Biomedical data were collected at 1 of 3 clinical research sites over a period of two days. On the second morning of the visit, participants provided a fasting blood sample for a lipid panel of total cholesterol, HDL-C, LDL-C, and triglycerides. The samples were initially stored in a − 60 °C to −80 °C freezer at each site, and then frozen serum (1-μl aliquots) were shipped on dry ice to Meriter Laboratories (Madison, Wisconsin) and stored at −65 °C. All assays were performed with a Cobas Integra analyzer (Roche Diagnostics, Indianapolis, Indiana). An enzymatic colorimetric assay was used for HDL-C, and triglycerides; LDL-C was derived using the Friedewald calculation [31]: for a limited number of respondents (1%) triglyceride levels were >400 mg/dl (milligram/deciliter); these observed values were replaced with 400 mg/dl to calculate the LDL-C level. From the biomarker project sample, HDL-C values ranged from 22 to 121 mg/dl; the interassay coefficient of variation (CV) was 2.2% to 2.3% and intra-assay CV 1.1% to 1.5%. Triglyceride values varied from 25 to 765 mg/dl; the interassay CV was 1.9% and intra-assay CV 1.6%. LDL-C computed values ranged from 16 to 283 mg/dl and the interassay CV was 10.11%. For the purpose of analysis, the HDL-C, LDL-C, and triglyceride variables were winsorized and log-transformed to normalize their distributions. Descriptive statistics for these variables and the other Time 2 measures are shown in Table 1.

**Table 1:** Descriptive statistics of time 2 measures

Type	Variable Description	Range	Mean or % (SD)
Demographics	Education	1–12	7.7 (2.4)
	Age	34–84	55.3 (11.8)
	Gender (female)	0–1	55%
Meds and Diabetes	Taking blood pressure meds	0–1	35%
	Taking cholesterol meds	0–1	30%
	Taking cortisol meds	0–1	4%
	Taking depression meds	0–1	15%
	Log of Homeostatic Model Assessment (HOMA) insulin resistance ((glucose X insulin) / 405)	−3.3 – 3.9	0.87 (0.77)
Health Behaviors	Currently smoking cigarettes	0–1	11%
	Alcoholic drinks in last month	0–240	13.3 (23.7)
	Exercise: 20 min 3 times/week	0–1	79%
	Healthy eating index (fruit/vegs, whole grains, white-to-red meat ratio)	0.9–5.8	2.5 (0.9)
	BMI	14.9–57.4	29.1 (5.9)
Blood lipids^a^	HDL-C (mg/dL)	1.34–2.08	1.72 (0.14)
	Triglycerides (mg/dL)	1.39–2.54	2.05 (0.22)
	LDL-C (mg/dL)	1.20–2.30	2.00 (0.15)

^a^Values have been winsorized and log-transformed

### Characterizing longitudinal trajectories of well-being

**Fig. 1:**
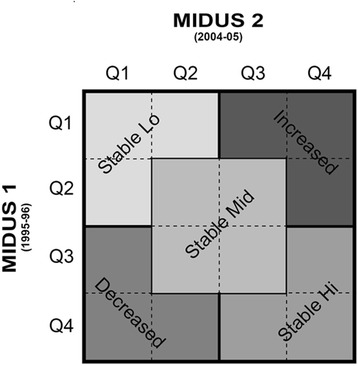
Typology of Longitudinal Trajectories of Well-Being (Quartiles)

Following a typology previously created [19], MIDUS 1 scores (using quartile cuts) on psychological well-being were cross-classified with scores at MIDUS 2 (using quartile cuts). This cross-classification produced a matrix containing 16 cells that were then divided into five categories (see Fig. 1). This matrix differentiated between respondents who were stable, albeit at differing levels: low, medium, high, and those who were decreasing or increasing in well-being, which required upward or downward change of 2+ quartiles.

Relative to longitudinal change scores, the typology has the advantage of addressing both magnitude and direction of change, while also accounting for individual differences in baseline levels). Per the index of reliable change [18], 90% to 94% of MIDUS respondents did not meet criteria for reliably increasing or decreasing on any scale of well-being over time. However, the typology employed slightly less stringent criteria for defining stability. Thus, across the six dimensions of well-being, 78% to 83% of respondents were classified as stable and were fairly equally divided between low, medium, and high levels of stability. These varieties of stability were compared with respondents classified as changing (increasing or decreasing). The portion of such cases ranged from 17% to 22% across the six scales of well-being.

### Statistical analyses

Hierarchical multiple regression analyses were performed using the three lipid variables as outcomes. In the first model, the demographic measures and a set of four dummy-coded variables (Stable Lo, Stable Mid, Decreasing, Increasing) representing the different longitudinal trajectories of psychological well-being were entered, with Stable Hi serving as the reference category. In the second model, control variables representing medication use and a measure of insulin resistance were entered. In the third model, health behaviors were entered. This hierarchical procedure allowed the examination of the effects of cross-time psychological well-being on lipid levels in conjunction with differing categories of control variables. By entering the trajectory variables in the first model, subsequent models suggested possible mediating mechanisms—particularly the health behaviors in model 3—through which psychological well-being may affect lipids.

Because the MIDUS biomarker sample included some twin pairs, assumptions of independent observations are violated. To address whether these familial dependencies biased the results, mixed effects models with random intercepts for family clusters were used to re-run analyses. The coefficients from these analyses were used to graph significant results from the stepwise regression procedure.

## Results

Table 1 shows descriptive statistics for the MIDUS 2 variables used in this analysis (demographics, medications and insulin resistance, health behaviors, lipids). The sample was divided fairly equally between men and women who were 55.3 years of age on average (range: 34 to 84). About a third of respondents were taking blood pressure or cholesterol medications. Further health information not included in the table: 36% of respondents met criteria for metabolic syndrome, 33% showed blood hypertension, 10% were diagnosed with diabetes, and 11% reported previous cardiovascular disease. With regard to blood lipids, low HDL-C (< 1.03 mmol/L (millimoles per litre) or 40 mg/dl), high triglycerides (> 1.69 mmol/L or 150 mg/dL) and non-optimal LDL-C (> 2.58 mmol/L or 100 mg/dL) were present in 20%, 28%, and 12%, respectively.

### Regression analyses: Prediction of lipid outcomes from well-being trajectories

Table 2 shows the standardized regression coefficients and *p*-values for the Stable Lo and Stable Mid trajectories relative to Stable Hi (reference category) that achieved statistical significance in Model 1. No analyses of trajectories for other aspects of well-being or for decreasing or increasing profiles remained significant in Model 3 and thus are not shown.

**Table 2:** Stepwise Multiple Regression Analysis of Longitudinal PWB Trajectories Predicting HDL-Cholesterol, Triglycerides, and LDL-Cholesterol

	Model 1^a^		Model 2		Model 3	
	Β	*P*	Β	*P*	Β	*p*
HDL-Cholesterol						
Environmental Mastery						
Stable Lo	−.149	.000	−.123	.000	−.110	.000
Stable Mid	−.089	.011	−.075	.021	−.077	.013
Personal Growth						
Stable Lo	−.085	.000	−.068	.023	−.046	.105
Stable Mid	−.088	.008	−.055	.070	−.047	.105
Purpose in Life						
Stable Lo	−.071	.034	−.066	.031	−.044	.140
Self-Acceptance						
Stable Lo	−.105	.002	−.087	.006	−.058	.053
Triglycerides						
Environmental Mastery						
Stable Lo	.170	.000	.132	.000	.121	.000
Stable Mid	.097	.011	.077	.023	.071	.034
Self-Acceptance						
Stable Lo	.121	.001	.092	.005	.075	.022
LDL-Cholesterol						
Environmental Mastery						
Stable Lo	.068	.082	.082	.024	.069	.057

^a^Model 1 included longitudinal trajectories of PWB and age, education, and gender; Model 2 added medication use (blood pressure, cholesterol, cortisol, depression) and insulin resistance; Model 3 added health behaviors (smoking, alcohol, healthy eating, exercise, BMI). Only results that achieved statistical significance for Stable Lo and Mid trajectories in Model 1 are shown

**Fig. 2:**
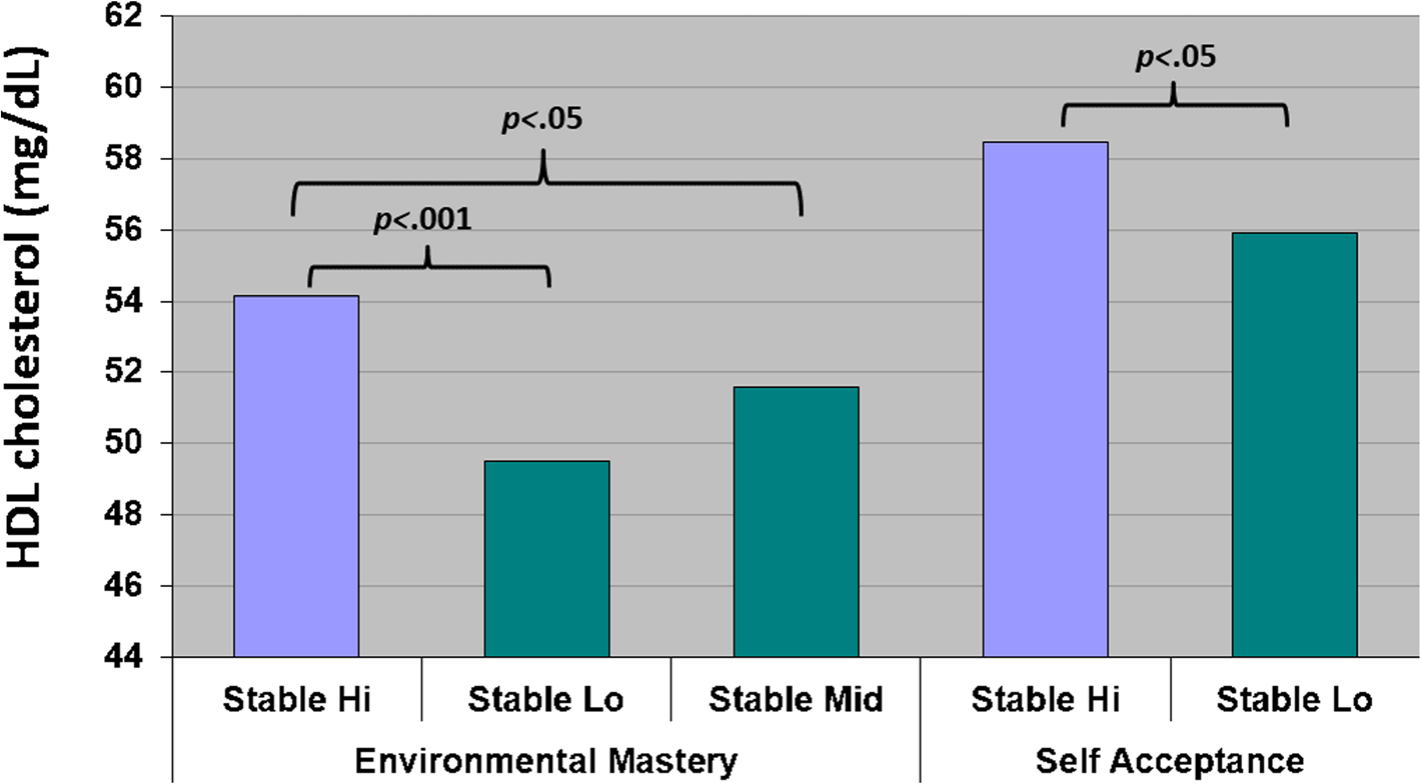
Longitudinal Trajectories of Well-Being and HDL in Model 3

For the prediction of HDL-C, analyses revealed that respondents with Stable Hi trajectories had significantly higher levels of HDL than those with Stable Lo trajectories for environmental mastery and self-acceptance. These effects are illustrated in Fig. 2. We note that although significant effects of psychological well-being trajectories for personal growth and purpose in life were initially evident in predicting HDL-C, such effects were not significant in Model 3.

Some covariates also significantly predicted HDL levels in Model 3 (effects not shown). Significantly higher (better) HDL-C levels were observed in older and female respondents. Respondents taking blood pressure medication, having higher BMI, or being regular smokers had lower HDL-C levels, while higher alcohol consumption, and reduced insulin resistance were associated with higher HDL-C levels.

**Fig. 3:**
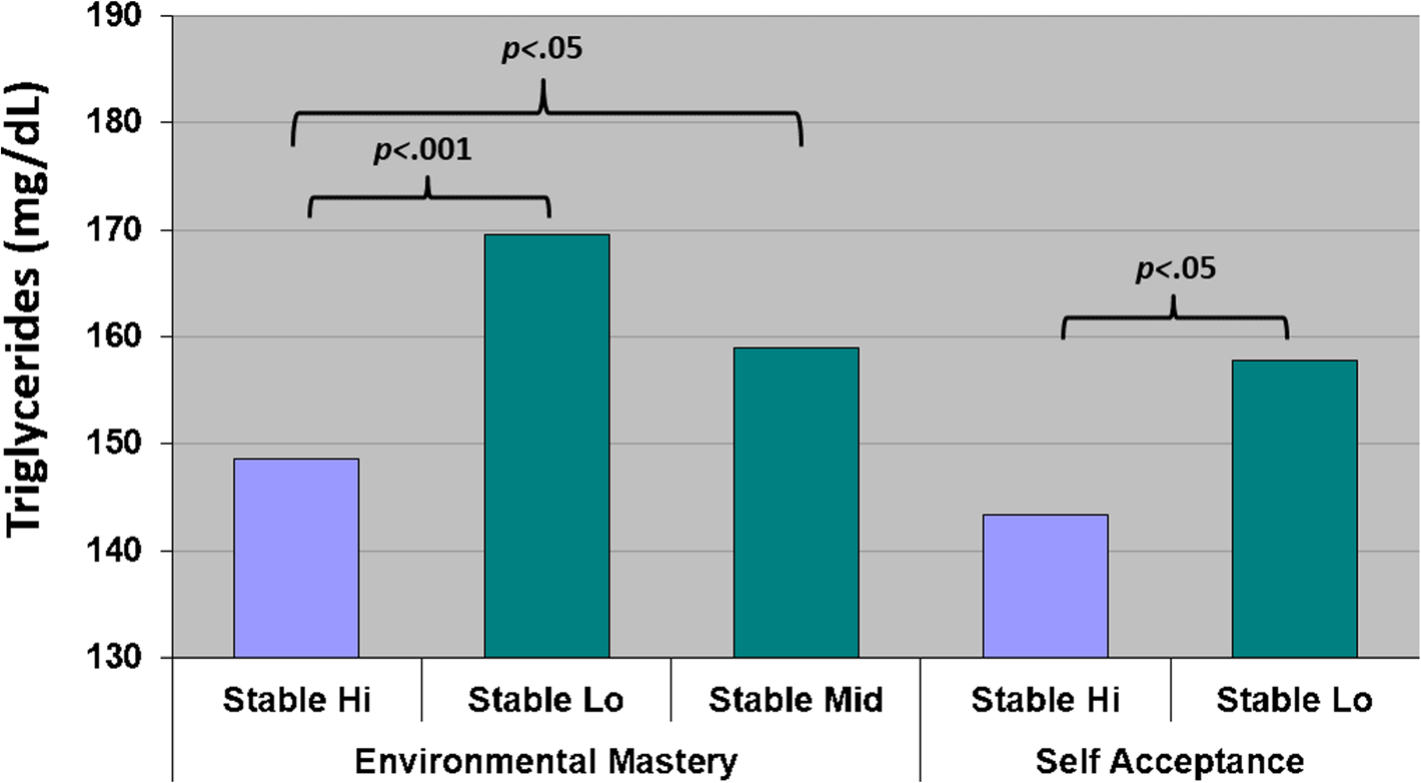
Longitudinal Trajectories of Well-Being and Triglycerides in Model 3

For the prediction of triglycerides, analyses also showed that respondents with Stable Hi trajectories in environmental mastery and self-acceptance had significantly lower levels of triglycerides than those with Stable Low trajectories (and Stable Mid trajectories for environmental mastery). These effects are illustrated in Fig. 3. Regarding covariates, female respondents had significantly lower levels of triglycerides, as did those with a better diet. Respondents with greater insulin resistance and higher BMI had elevated triglyceride levels.

For the prediction of LDL-C, an initial effect of Stable Hi versus Stable Lo trajectories of environmental mastery was marginally significant, but was attenuated after adjusting for biological dependencies in the data. Nonetheless, the difference in LDL-C levels between Stable Hi and Stable Lo has clinical or functional consequences that can be expressed in a meaningful metric (see discussion below).

Among covariates in Model 3, medication use (blood pressure and cholesterol) was significantly associated with lower LDL-C levels, as was a better diet. Increased insulin resistance, smoking and a higher BMI, however, were associated with elevated levels of LDL-C.

## Discussion

Well-being has been linked with a range of biomarkers and health outcomes [13–15, 17]. In this emerging research area, some studies, mostly cross-sectional, have linked between well-being and blood lipids (reviewed in [9]). With regard to hedonic well-being, greater happiness was associated with lower levels of total cholesterol [1], while positive affect correlated with higher HDL-C, but not the ratio of total/HDL-C [32]. With regard to eudaimonic well-being, which emphasizes proactive engagement in life and is the focus of this study, a cross-sectional analysis of older women reported that personal growth was inversely related to total/HDL-C ratio [32]. Personal growth and purpose in life were also positively associated with HDL-C in the same study. Additionally, low sense of coherence in middle-aged women was correlated with lower HDL cholesterol and higher triglyceride levels [33]. Most recently, greater optimism was associated with a healthy lipid profile in MIDUS adults [5].

However, cross-sectional studies linking well-being and lipids are inherently limited, although studies taking a long-term approach are few in number. In one study, self-sufficiency during adolescence was associated with lower total cholesterol, but not HDL-C, in adult men, after adjusting for lifestyle and biological factors [34]; the effects were not evident in adult women, however. Short-term longitudinal studies of optimism and other well-being measures (e.g., vitality) have provided mixed evidence regarding lipids [35, 36]. The current study, although lacking longitudinal assessment of lipids, offers comprehensive measurement of eudaimonic well-being evaluated twice over a 9–10 year period in a national sample of U.S. adults. Thus, it was possible to characterize respondents with regard to their longitudinal trajectories of well-being and then use these characterizations to examine variation in lipids, assessed for the first time during the 2nd wave of the study. Adding to the novelty of the inquiry was that most respondents showed high stability in their reported well-being over time, albeit at notably different levels [19]. The central question of this inquiry was thus whether those with persistently high levels of eudaimonic well-being would show better lipid profiles at follow-up compared to those with persistently low levels of well-being.

Respondents with advantaged (persistently high) levels of environmental mastery and self-acceptance, indeed, had significantly higher levels of HDL-C and lower levels of triglycerides than those reporting disadvantaged (persistently low) levels in these two aspects of well-being. Such outcomes suggest that high levels of these two aspects of well-being may be protective against atherosclerotic cardiovascular disease, given its predictive association with this less atherogenic lipid profile. Indeed, the 2–5 mg/dl increase in HDL-C observed in subjects with advantaged versus disadvantaged well-being predicts a 4–10% range in reduction of coronary heart disease [37], similar to the influence expected from lifestyle interventions and lipid-modifying drugs with low-to-moderate effects on HDL-C levels. These effects were evident after adjusting for demographic and health factors as well as accounting for biological dependencies in the data.

The initial analysis (Model 1) showed that time trajectories in different psychological well-being components (environmental mastery, personal growth, purpose in life, and self-acceptance) were associated with differences in HDL-C or triglyceride levels, such effects only remain significant for environmental mastery and self-acceptance in Model 3. These findings suggest that covariates used in Model 2 and 3 are possibly involved as mediators between well-being parameters and HDL-C and triglyceride levels. Why was the effect of environmental mastery and self-acceptance on lipids not observed for other aspects of psychological well-being, such as purposeful life engagement, positive relationships with others, and personal growth – many of which have been linked to biological risk factors as well as morbidity and mortality outcomes (see [17] for a review of prior findings)? We hypothesize that in the same way that different physiological systems matter for different disease outcomes, it may also be the case that different psychological factors are relevant for different physiological consequences and/or biomarkers. For instance, purpose in life and positive interpersonal relations -but not other components of eudaimonic well-being- correlated with interleukin-6, a well-known inflammatory marker [38]. In addition, only purpose in life has been associated with better glucoregulation (i.e., lower glycosylated hemoglogin (HbA1c)) in Japanese [39], but not American [40] subjects. Furthermore, personal growth was the only eudemonic well-being variable that predicted reduced risk for metabolic syndrome both cross-sectionally and longitudinally [41]. As a whole, these emerging findings from the present study as well as previous investigations point to quite differentiated patterns of effects, in which distinct components of eudaimonic well-being are aligned with different health outcomes, including biological risk or protective factors. Continuing inquiries are needed to establish the replicative consistency of these observed patterns.

It is important to note that longitudinal trajectories of psychological well-being were not linked to variations in LDL-C levels. Such results converge with findings from previous investigations on other positive psychosocial resources, such as sense of coherence [33] and optimism [5], which have also showed links with HDL-C and triglycerides but not LDL-C. Relatedly, studies of personality traits such as conscientiousness and impulsivity have also been more consistently associated with HDL-C and triglycerides rather than with LDL-C or total cholesterol [42]. This convergence with previous reports strengthens confidence in the findings reported in our analysis.

## Conclusions

These results as well as previously reported associations between positive psychosocial resources and cardiovascular disease associations (reviewed in [9, 43, 44]) have possibly important implications for the development of novel non drug-based strategies for management of residual cardiovascular risk remaining after intensive LDL-C lowering with statin therapy. For example, “well-being therapy” has been shown to be effective in treating individuals with psychological disorders, such as depression or anxiety [45, 46]. The central focus of such therapy is to increase patients’ experiences of psychological well-being. Outside the clinical context, preventive interventions have been conducted in school and community settings to increase the well-being of adolescents and older persons [47–50]. The take-home message is that aspects of psychological well-being, which increasingly show protective benefits (reduced profiles of biological risk, reduced morbidity, extended longevity), are modifiable. Through targeted interventions, they can be enhanced, thereby increasingly the proportion of individuals in the general population who might experience the health benefits associated with heightened psychological well-being.

Limitations of the current inquiry must be considered. Although the analysis benefited from longitudinal assessments of psychological well-being, lipids were measured only at a single point in time. Future inquiries of long-term assessments of both well-being and lipids are needed to clearly discern patterns of causal influence between the two domains of assessment. As noted above, the obtained patterns were evident for only select aspects of psychological well-being and two of three lipid assessments. The replicative consistency of these effects needs to be examined. Of particular importance for future inquiry are studies that address the mechanistic pathways through which reported levels of well-being might influence lipids, such as daily health practices (e.g., healthy eating, regular exercise regimens, not smoking). Although based on a national sample of U.S. adults, this inquiry is also limited to a single sociocultural context. Whether the findings generalize to other countries with different dietary practices and perhaps different cultural formulations of well-being are important future directions.

Despite such limitations, linkages of eudaimonic well-being with various physical, health and/or disease outcomes are growing in scientific prominence and include findings that extend beyond the cardiovascular system, such as lower prevalence of neurodegenerative disorders, overall healthy aging, and longer life expectancy [15, 17]. Interestingly, higher HDL-C levels have been present across diverse outcomes, including studies of memory [26], dementia [27], and increased longevity in various populations [28]. A better blood lipid profile, particularly higher HDL-C, may be key in mediating how psychological well-being impacts disease, health and length of life. Whether these linked well-being/lipid profiles are associated with less disease is important and will be examined as additional longitudinal data on the MIDUS sample accumulates. Further research is also required to explicate potential underlying HDL-dependent mechanisms, such as anti-oxidant and anti-apoptotic properties of HDL particles beyond HDL-C levels and reverse cholesterol transport [51–54].

In summary, these findings add multiple novel advances to the emerging literature linking psychological well-being to lipid profiles: (1) the research utilizes a national longitudinal sample of adults to examine these relationships, thereby strengthening the generalizability and potential applicability of the findings; (2) the analysis employs longitudinal assessments of psychological well-being to predict baseline lipid profiles – all prior research has relied on cross-sectional data to connect these two domains; and (3) the approach to the cross-time measurement of psychological well-being distinguishes between cumulative cross-time profiles – specifically, between those who are persistently high versus persistently low in their experiences of psychological well-being. The central findings are that those with persistently high well-being show better lipid profiles (i.e., higher HDL levels, lower triglycerides) compared to those with persistently low well-being. No prior research has linked longitudinal assessments of well-being to lipids in a national sample of adults.

## Abbreviations

BMI: Body Mass Index
CHD: Coronary Heart Disease
CV: Coefficient of Variation
HbA1c: glycosylated hemoglobin
HDL: High Density Lipoproteins
HDL-C: High Density Lipid Cholesterol
LDL: Low Density Lipoproteins
LDL-C: Low Density Lipid Cholesterol
mg/dl: milligrams per deciliter
MIDUS: Midlife in the United States
mmol/L: millimoles per Liter
PWB: Psychological Well-Being
